# Biomechanical Evaluation of Decellularized and Crosslinked Corneal Implants Manufactured From Porcine Corneas as a Treatment Option for Advanced Keratoconus

**DOI:** 10.3389/fbioe.2022.862969

**Published:** 2022-04-14

**Authors:** Abby Wilson, John Jones, John Marshall

**Affiliations:** ^1^ UCL Mechanical Engineering, London, United Kingdom; ^2^ Institute of Ophthalmology, UCL, London, United Kingdom; ^3^ Laser Optical Engineering Ltd., Donington, United Kingdom

**Keywords:** keratoconus, implants, crosslinking, decellularization, interferometry, xenotransplantation, corneal biomechanics

## Abstract

Currently corneal transplantation is the main treatment for late-stage keratoconus; however, transplantation procedures are accompanied by significant risk of post-surgical complications; this in addition to supply limitations imposed by a worldwide shortage of human donor corneas, has driven the development of alternative therapies. One such therapy is the use of corneal implants derived from porcine corneas (Xenia^®^, Gebauer Medizintechnik GmbH, Neuhausen, DE). In contrast to human donor tissue, these implants can be produced on demand and due to the processes used pose no risks for host-immune rejection. Their use has already been demonstrated clinically in patients for preventing the progression of topographic changes in keratoconus whilst improving visual acuity. The implants are derived from natural tissue and not standardised synthetic material, whilst this likely reduces the risk of issues with bio-incompatibility, there is inevitably variability in their intrinsic mechanical properties which requires investigation. Here, speckle interferometry is employed to examine the biomechanical properties, in response to physiologically representative forces, of native porcine corneal tissue prior to processing and after a proprietary 4-stage process involving decellularization, washing, compression and crosslinking. The control lenticules had an average Young’s modulus (E) of 11.11 MPa (range 8.39–13.41 MPa), following processing average E of the lenticules increased by 127% over that of the unprocessed tissue to 25.23 MPa (range 18.32–32.9 MPa). The variability in E of the lenticules increased significantly after processing suggesting variability in the propensity of the native tissue to processing. In summary, it is possible to produce thin (<90 µm) lenticules from porcine corneas with enhanced stiffness that are effective for treating late-stage keratoconus. Due to the observed variability in the responses of lenticules to processing, interferometry could be a useful technique for ensuring quality control in commercial production via biomechanical screening.

## Introduction

There is a large variability in the reported incidence of keratoconus (Gokhale, 2013) with one publication showing it to be as high as 1 in 375 people worldwide (Godefrooij et al., 2017a). Diagnosis is often made after significant topographic changes have occurred as a result of the progressive biomechanical decompensation that takes place due to the presence of localised abnormalities in the stroma (Roy et al., 2013). The incidence of mild ‘sub-clinical’ keratoconus may be far in excess of these reported values, but it remains largely undetected due to the sensitivity restrictions of currently available diagnostic tools (Wilson and Marshall, 2020). On diagnosis, patients often have perceivable visual abnormalities which without treatment progressively worsen over time. Treatment options vary from contact lenses, intra-ocular lenses (IOL’s), topography-guided keratoplasty, corneal crosslinking (CXL) (Andreanos et al., 2017) and in more advanced-stages of the disease—corneal transplant surgery in the form of penetrating keratoplasty (PK) or the generally preferred—deep anterior lamellar keratoplasty (DALK) (Parker et al., 2015; Andreanos et al., 2017) due to its advantages with regards to maintaining the patient’s own endothelium.

Until recently corneal transplant surgery has been the only effective treatment for late-stage keratoconus, however it has several constraints in terms of both procedural challenges and a limited tissue supply bank. Transplantation procedures are often long and difficult, relying upon highly skilled and experienced surgeons. They carry significant risk of post-operative complications including infection, glaucoma, cataracts, and host immune-rejection (Koo et al., 2011) with reported rejection rates ranging from 2% up to 68% (Andreanos et al., 2017). Visual outcomes take several months to stabilise and are generally sub-optimal, with the procedures themselves contributing to astigmatism resulting in patients requiring either complex glasses or contact lenses for refractive correction post-surgery (Andreanos et al., 2017). All these factors contribute to a high economic cost for disease management, alongside a cost to the patient both in monetary terms and with regards to their quality of life. Furthermore, with over 12.7 million people currently waiting for corneal transplant, there is a significant shortage of donor tissue worldwide (Williams and Muir, 2018) with around 185,000 procedures being performed per year, meeting the needs of approximately 1 in every 70 patients (Gain et al., 2016). The United Kingdom falls short by approximately 1,500 corneas every year (Gaum et al., 2012), leading to long waiting times for patients, again impacting their quality of life and ability to work whilst living with severe visual impairment. In the United Kingdom, the economic cost of visual impairment per person may be in excess of £10,000 per annum (Pezzullo et al., 2018), hence, improving current treatment options for late-stage keratoconus and solving the restrictions posed by the current tissue shortage is of high importance.

To address some of these issues, recently, Bowman’s layer transplantation (BLT) surgery, where isolated human donor Bowman’s layer is inserted into a mid-stromal pocket in keratoconic corneas (Dragnea et al., 2018), has been attempted to address advanced cases of keratoconus, with some success (Van Dijk et al., 2014; van Dijk et al., 2015). It has advantages over traditional corneal transplant surgery as it is a suture-less procedure and the tissue is acellular, reducing recovery times and graft rejection rates (Dragnea et al., 2018). It also makes use of corneas that are otherwise unsuitable for transplantation due to poor endothelium quality, however, still relies on a limited tissue resource, and currently isolation of the Bowman’s layer is performed manually and is challenging with reported failure rates of up to 30% (García de Oteyza et al., 2019). Ideal treatment alternatives to corneal transplant surgery for treating both early and later-stage keratoconus are those that do not rely on human tissue supply, and this is where many research efforts are now focussed.

One treatment, aimed at reinforcing biomechanically compromised corneas, which has shown potential for treating late-stage disease (El-Massry et al., 2021) is Xenia^®^ corneal implants (Gebauer Medizintechnik GmbH, Neuhausen, DE). An increasing number of patients are now undergoing this treatment with several now with over 12 months follow-up (El-Massry et al., 2021).

Xenia^®^ corneal implants are created by processing porcine corneal stroma. Due to several structural (Sharifi et al., 2019; Subasinghe et al., 2021) and biomechanical similarities (Zeng et al., 2001; Elsheikh et al., 2008) of porcine and human corneal tissue, the baseline material from which the Xenia^®^ implants are derived, although not identical, more closely resembles the properties and structure of the human cornea in contrast to implants manufactured from synthetic alternatives. Currently available synthetic alternatives have shown poor long-term success rates, which is thought in-part to be due to their bio-incompatibility. Common complications include corneal melt around the prosthetic and the development of glaucoma with long-term adjunct therapies required to try to prevent these conditions (Akpek et al., 2014; Nonpassopon et al., 2020). Hence a drive towards the development of implants centred around natural and structurally similar materials.

To form Xenia^®^ lenticules, tissue obtained from the central anterior portion of porcine corneas, comprising the Bowman’s layer (the presence of which has recently been confirmed (Hammond et al., 2020) after some controversy over its existence in pigs) and most anterior portion of stroma, is subjected to a four-stage process. This process involves: de-cellularisation which remove cells, antibodies and antigens negating the risk of rejection by the patient’s immune system; washing; compression; and crosslinking to reduce the thickness and increase the stiffness of the lenticule relative to normal corneal tissue, which is important for stabilisation and reshaping of the keratoconic cornea. As with BLT, the Xenia^®^ implant is inserted into the cornea via a pocket in the stroma that is created by a femtosecond laser, once inserted it acts as a splint to increase corneal stiffness and resistance to intraocular pressure, which in turn has been shown to have positive effects on corneal topography (El-Massry et al., 2021). Overall, the Xenia^®^ implant has several significant advantages over conventional corneal transplant surgery; it is a minimally invasive procedure requiring only topical anaesthetic enabling it to be performed in an out-patient setting, significantly reducing treatment costs and time; it is suture-less meaning faster recovery, lower risk of complications and better visual outcomes. In addition, because Xenia^®^ implants can be used to slightly increase the thickness of the cornea they have the potential to be used to enable other treatments such as corneal crosslinking (CXL) in patients with thinner corneas which may act to further improve stability and visual outcomes (El-Massry et al., 2021).

Further to the aforementioned benefits, with future research these implants may have the potential to be tailored to individual patients to optimise refractive outcomes without the need to remove tissue from the cornea as in traditional laser refractive surgery procedures, which can have negative implications for overall biomechanics (Wolle et al., 2016; Fernández et al., 2018), and increase the risks associated with potential follow-up procedures such as CXL. Hence, they could ultimately prove to be an effective treatment option for both early and late-stage keratoconus.

With biomechanical abnormality being central to the progression of topographic abnormality and visual deterioration in keratoconus, most treatments are centred around preventing the consequences of this. Hence, biomechanical evaluation of the effects of potential treatment options plays a key role in their development and clinical adoption.

A better understanding of the biomechanics of Xenia^®^ implants and their effect on the biomechanics of the host cornea is required to enable optimisation of this treatment in terms of safety, efficacy and the quality of visual outcomes. The biomechanics of the cornea and its resistance to intra-ocular pressure (IOP) is what governs its unique shape and hence refractive properties, as such it is important to understand how the insertion of an implant affects the whole system. Furthermore, as the implants are formed from material derived from biological tissue, a greater understanding of the potential variability of biomechanical properties across implants is required, in addition to a better understanding of the individual biomechanics of the system into which it is inserted. Pilot studies of CXL for both keratoconus (O'Brart et al., 2015) and myopia (Juthani and Chuck, 2021) have shown that there is variability between patients with regards to their response to crosslinking treatments, hence the same is likely to be true of the porcine tissue from which the lenticules are formed. With a better understanding of these factors, it may be possible to accurately control the biomechanical properties of individual implants and in doing so manufacture implants with customised properties for an individual’s cornea, or to quantify and standardise the properties of implants used in surgery.

Understanding tissue biomechanics in response to physiologically representative loads is important when dealing with biological tissues, such as the cornea, as they possess viscoelastic properties, resulting in different properties in response to loads of different magnitudes and loading rates (Elsheikh et al., 2007). Since it is of importance to understand how the materials will behave *in vivo*, physiological loads must be replicated. Recently, speckle interferometry has been shown to be a useful method for examining the load-deformation response of corneal tissue in response to pulsatile loads representative of those that occur over a normal cardiac cycle (Wilson et al., 2016; Wilson et al., 2020; Wilson et al., 2021). This empirical data highlighted that speckle interferometry could be a useful tool to assess the load deformation response of Xenia^®^ implants as it has high sensitivity enabling deformation to be quantified with an accuracy of 10s of nanometres, providing high resolution displacement maps in response to pressure changes equivalent to those that occur over a normal cardiac cycle.

The present study investigates the effects of processing (decellularization, washing, compression and crosslinking) on the mechanical strength of porcine cornea lenticules used for Xenia^®^ implants. This is achieved by using displacement speckle pattern interferometry (DSPI) to examine the load-deformation response of samples to pulsatile, physiologically representative, pressure variations.

## Methods and Materials

### Lenticules

Six control lenticules that had not been subjected to any processing and four processed lenticules were prepared for measurement, the lenticules were dissected from porcine corneas obtained from Schlachthof e.G., 71116 Gaertringen, DE. The lenticules were 9 mm in diameter and obtained from the anterior surface of the central porcine corneal stroma, with thicknesses between 190–210 µm prior to processing. Four of the specimens isolated from porcine corneas underwent a proprietary process (patent pending) at Gebauer Medizintechnik, GmbH, Neuhausen, DE involving 4 stages: decellularization, washing, compression and crosslinking. The process used resulted in a more than 50% reduction in the thickness of the lenticules to 90 µm.

### Experimental Set-Up and Measurement Principles

**FIGURE 1:**
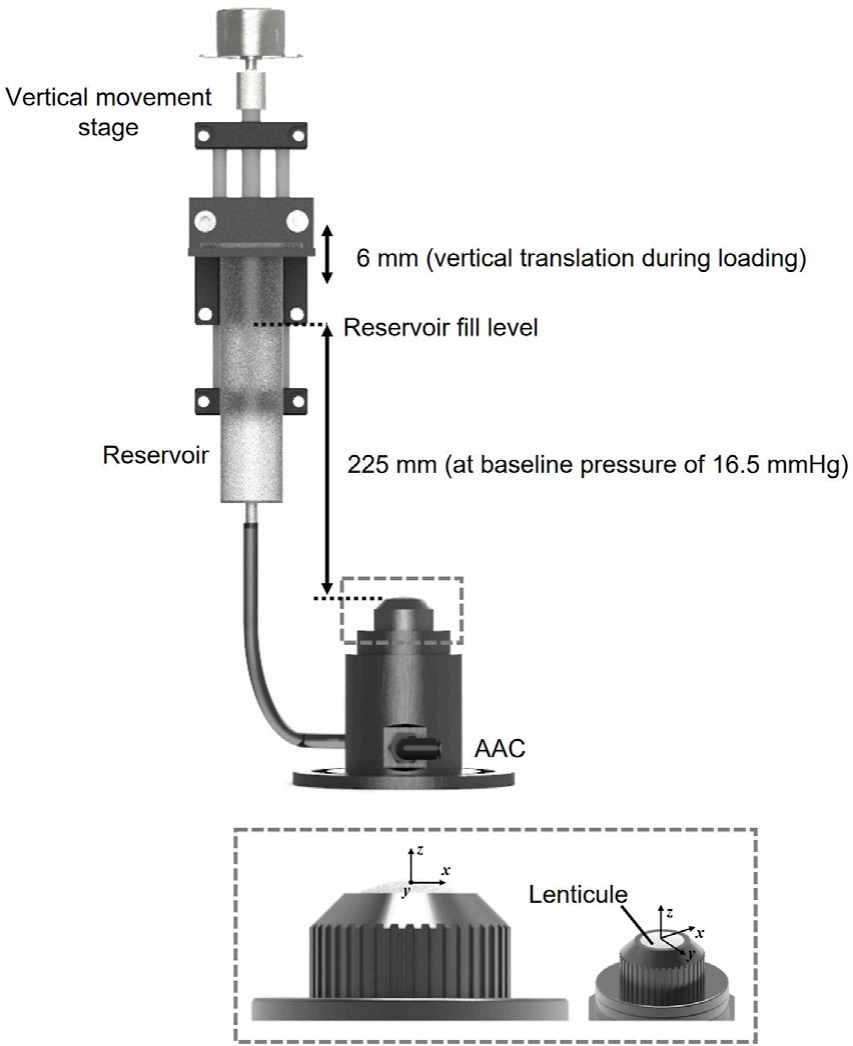
Diagram of loading system configuration and the custom artificial anterior chamber (AAC) with 7 mm aperture into which lenticules were clamped.

Prior to interferometric measurement lenticules were mounted into a custom-designed artificial anterior chamber as shown in Figure 1. The aperture of the chamber was 7 mm in diameter leaving a 1 mm boundary for clamping around the circumference of each of the lenticules. The chamber was attached to a reservoir via the inlet. The reservoir was mounted onto an automated vertical translation stage. Both the chamber and reservoir were filled with phosphate buffered saline (PBS) solution (Sigma-Aldrich, United Kingdom, ρ = 0.995 g/ml at 25°C) The height of the reservoir relative to the top surface of the lenticule was used to control the pressure in the chamber with the baseline pressure set at 16.50 mmHg which is representative of normal IOP in porcine corneas (Ruiz-Ederra et al., 2005). Pressure variations from this baseline pressure were achieved by increasing the height of the reservoir relative to the top surface of the lenticule.

**FIGURE 2:**
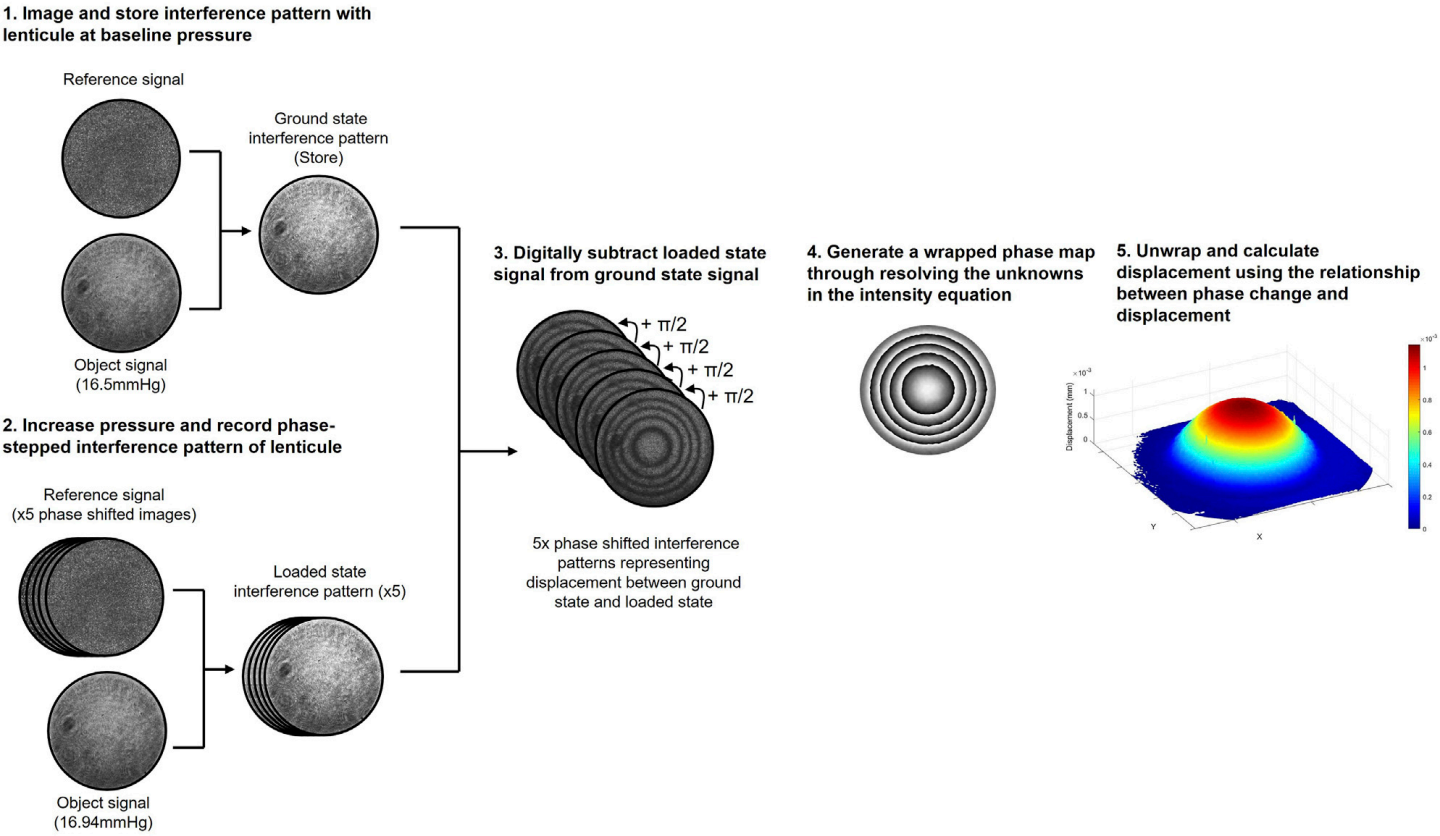
Diagrammatic summary of the DSPI data acquisition and processing procedure used to measure axial displacement.

The interferometer used for displacement measurements and the measurement principles (Wilson and Marshall, 2018) were identical to those described in detail in previous recent publications (Wilson et al., 2020; Wilson et al., 2021). Figure 2 provides a diagrammatic summary of the measurement principles of DSPI. Briefly, monochromatic, coherent light is used to illuminate the surface of a sample, the backscattered light from the surface is interfered with that of a reference beam (in this instance a portion of the illumination beam reflected from a mirror) and imaged through a lens by a camera. The resulting speckle pattern formed from the constructive and destructive interference of light waves from the object and reference portions of the beam is imaged and stored as a reference (ground state). Each speckle in this image can be considered as a unique data point with a specific intensity (I) as described by Eq. 1 (Petzing and Tyrer, 1998) and related to the phase difference (Δφ) of the object (o) and reference (r) wavefronts, which is proportional to the position of each specific point on the objects surface relative to the reference surface.

$$I=\;I_{r}+\;I_{O}+2\sqrt{I_{r}I_{O}}\cos\Delta \varphi$$ (1)

When the surface of the object moves in response to a stimulus the speckle pattern changes due to a change in the phase difference between the object and reference portions of the beam. Through digitally subtracting the speckle patterns that form as the surface of the object deforms from the reference (ground state) to the final (loaded state) we generate an interferogram composed of interference fringes, which with knowledge of the specific imaging set-up can be deciphered to determine components of surface displacement, the theory of which is described in greater detail elsewhere (Wilson and Marshall, 2018; Petzing and Tyrer, 1998).

Since, in the set-up described, the illumination and imaging were both configured normal to the surface of the sample (Supplementary Figure S1), the interference fringes related to axial surface deformation (Petzing and Tyrer, 1998). Due to the lenticule samples being initially flat, and the nature of the mounting and loading method used, axial deformation was expected to be significantly greater than lateral deformation in response to hydrostatic loading (Wilson et al., 2021) and therefore lateral deformation was not directly measured here. To generate quantitative information from the resulting interferograms, the reference beam was temporally phase-stepped during measurement, facilitating the generation of phase wrapped images. Temporal phase-stepping involves shifting the reference beam by a specific amount over a series of at least 3 images enabling all three unknowns in the intensity equation (Eq. 1) to be resolved and quantitative information to be extracted from the interferogram, the mathematics of which is described in greater detail in previous publications (Creath, 1988; Joenathan, 1994; Francis et al., 2010). For the measurements conducted in this study a 5-step phase shifting procedure and complimentary processing algorithm (Francis et al., 2010) was used with a phase step of π/2. A phase-unwrapping algorithm (Herráez et al., 2002) was used to remove the 2π discontinuities from the phase wrapped images (Wilson and Marshall, 2018) and generate smooth phase maps where the phase change $\left(\Delta \phi_{def}\right)$ was proportional to axial displacement (w), and could be calculated via Eq. 2 (Wilson and Marshall, 2018), where λ was the wavelength of the illumination source.

$$w=\;\Delta \phi_{def}\;.\;\frac{\lambda }{4\pi }$$ (2)

For the set-up used in this study (Supplementary Figure S1) illumination was via a diode pumped single-mode solid-state laser (*λ* = 532 nm) (06-DPL, Cobolt AB, Solna, SE), which was expanded and collimated to a diameter of 25 mm. The illumination beam was passed through a 50:50 beamsplitter with half directed towards the target surface (lenticule surface) and half towards a planar mirror attached to a piezo-electric transducer which was used to accurately phase step the reference beam by π/2 over a series of 5 images. The beams from the object and the reference were interfered, then imaged via a CMOS camera with a resolution of 1,296 by 972 pixels (CMOS Aptina MT9P031, Basler AG, Ahrensburg, DE) through a 12.5–75 mm zoom lens (C31204, Pentax, Tokyo, JP).

### Experimental Procedure

All lenticules were shipped to Loughborough, United Kingdom via 24-h delivery from Gebauer Mediziniechnik, 75242 Neuhausen, DE. During transportation and storage the lenticules were fully immersed in sample tubes filled with a trinity solution (50% glycerol, 30% water, 20% ethanol), chosen for the mixtures preservation properties and principal capability to be fully metabolised by cells of the human body. The sample tubes were enclosed in insulated packaging and surrounded by ice packs. On arrival the lenticules were stored at 4°C prior to measurement. Pre-mounting the lenticules were transferred into water and subjected to 30 min of mechanical shaking to facilitate the dilution of the trinity present inside the lenticules from the transportation and storage steps. Following this the lenticules were mounted centrally into the chambers, a light coating of white powder (Sphericel 110P8, Potters Ind. LLC, PA, United States) was applied to the surface of each of the samples. This coating was necessary to generate an adequate signal from the surface. Due to its particulate nature, the coating had no stiffness and moved with the underlying surface, therefore having no effect on the measured deformation of the lenticule when subjected to loading. The lenticules were set under a baseline pressure of 16.50 mmHg where they were rested for 20 min to stabilise under the initial pressure prior to the initiation of measurement. Following this each of the lenticules was subjected to 20 repeated loading cycles where the pressure was increased and then subsequently decreased by 0.44 mmHg in a pulsatile manner. A 3-s pause was programmed between cycles and for each cycle the reference image was captured at the baseline pressure (16.50 mmHg) with the loaded image captured at the highest pressure of 16.94 mmHg. The reason for this specific pressure increase of 0.44 mmHg was to optimise the number of interference fringes generated across samples to maximise the signal to noise ratio in the resulting images. During experimentation, one of the processed lenticules was damaged during the mounting stage and was therefore discounted for analysis.

### Data Processing

Post-measurement, the data sets from each lenticule were visually assessed and those with obvious noise or phase-stepping errors (evident from the quality of the fringes in the phase-wrapped images) were removed. All remaining data was imported into Matlab (MathWorks Inc., United States). Phase data from each lenticule was averaged and displacement across the full sample was calculated using the relationship between axial displacement and measured phase change as described in Eq. 2.

The deformation of the lenticules indicated relative uniformity in mechanical properties across each of the samples, as the fringes were close to circular with relatively even spacing which is what would be expected from a sample with spatially homogenous bulk material properties. Due to this the mechanical stiffness, in terms of Young’s modulus (E) of the samples could be estimated from the central (maximum) displacement (w_centre_) via Eq. 3 (Young and Budynas, 2002), where ΔP is the pressure change (0.44 mmHg, 59 Pa), R is the initial sample radius of curvature (7.8 mm, estimated as the average human corneal radius of curvature), t is sample thickness (0.2 mm unprocessed, 0.09 mm processed), and υ is corneal Poisson’s ration (0.42).

$$E=\;\frac{\Delta P\;.\;R^{2}}{2\;.\;w_{centre}\;.\;t}\;\left(1\;-\;\upsilon \right)$$ (3)

This calculation of stiffness is based on the following assumptions; the boundary conditions impose a pinned edge, the lenticule acts as a membrane where bending stiffness can be considered negligible and horizontal displacement is zero.

## Results

**FIGURE 3:**
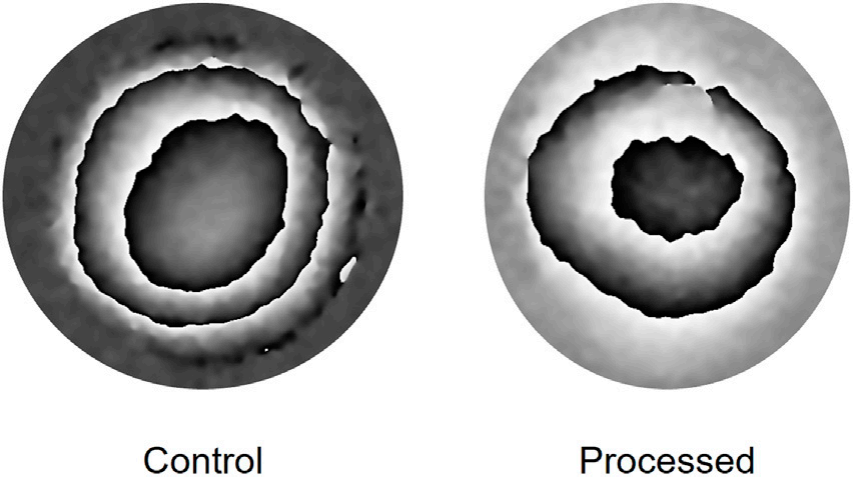
Representative phase wrapped images obtained when imaging the deformation of a lenticule over a pressure increase from 16.50 to 16.94 mmHg.

A representative wrapped fringe pattern from one of the control (unprocessed) and one of the processed lenticules is shown in Figure 3. The fringes were found to be relatively circular which would indicate x-y homogeneity in mechanical properties, however there were slight deviations from circular fringes evident, and this could be representative of slight variations in the mechanical properties of the superior-inferior and nasal-temporal axis of the corneas from which the samples were derived.

**FIGURE 4:**
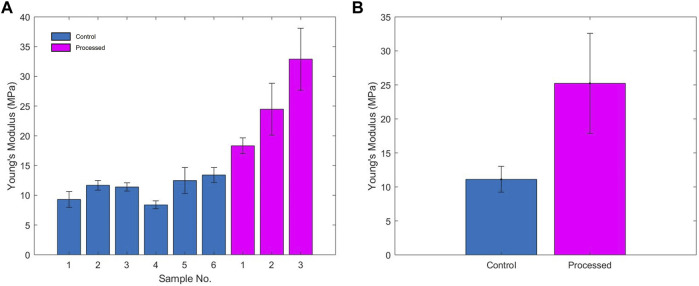
**(A)** Young’s modulus of each of the samples; **(B)** average Young’s modulus of unprocessed and processed samples.

The mechanical stiffness values that were derived from the data obtained during interferometric testing of each of the lenticules is summarised in Table 1 and in Figures 4A,B. One of the processed lenticules was damaged during testing and was therefore discounted in the analysis.

**TABLE 1:** Calculated Young’s modulus of unprocessed and processed samples.

Calculated Young’s modulus (MPa)	
Control Samples	Processed Samples
9.31±1.31	18.32±1.32
11.68±0.82	24.47±4.34
11.40±0.69
8.39±0.66	32.9±5.23
12.49±2.15	
13.41±1.25	

The control lenticules had an average Young’s modulus (E) of 11.11 MPa (range 8.39–13.41 MPa) compared to the processed lenticules 25.23 MPa (range 18.32–32.9 MPa). Overall processing of the samples resulted in an average increase in estimated Young’s modulus of 127% above that of the unprocessed tissue. Variability in the stiffness of the samples was greater between the processed samples with a range of 14.58 vs. 5.02 MPa in the unprocessed lenticules.

## Discussion

The purpose of these experiments was to evaluate the mechanical effects of the process used to produce Xenia^®^ corneal implants. Although measurement in this initial investigation was limited to a small number of samples, it was evident that processing of the lenticules resulted in a significant increase in stiffness by on average 127% over the unprocessed tissue that is initially extracted from the central anterior porcine corneal stroma. This increase in relative stiffness when compared to normal corneal tissue is important when considering the potential treatment of keratoconus as it means the implant could act as effective splint to counteract bulging and weakness of the keratoconic cornea into which it is inserted.

Further to the increase in mechanical stiffness, there were several interesting observations from this initial measurement data set. Firstly, the slight deviation from circular fringe distribution seen in the interferograms of the control samples indicates slight differences in mechanical properties with respect to different axes of the cornea. The actual axes of the tissue relative to the axes of the cornea from which it had been extracted were not identified in this study. This slight difference in mechanical properties and deviation from circular fringes was predicted from previous studies on the biomechanics of the whole cornea (Boyce et al., 2008; Wilson et al., 2020; Wilson et al., 2021), where it has been shown that mechanical properties are not homogenous across the tissue and particularly with respect to the superior-inferior and nasal-temporal axis. The heterogeneity of the mechanical properties of the native cornea could be an important consideration when manufacturing these implants. To keep properties as consistent as possible it will be important to take the tissue for the implant from the same position in each given cornea. However, as only small deviations were seen from circular fringes in the processed implants, during transplantation it is likely not to be critically important to know the exact orientation of the implant relative to the cornea from which it was removed.

A further interesting observation was that the variability in the calculated stiffness of the processed lenticules was significantly greater than in the control tissue. This may have arisen due to the combined effect of differences in initial tissue mechanical properties between different corneas and differences in their ability to respond to the four processing stages. For example, many crosslinking studies have shown that the response to crosslinking is patient-dependent with some individuals being high-responders and others, low-responders (Godefrooij et al., 2017b). The reasons for this are difficult to identify as there are likely to be several contributing factors and therefore it is difficult to control. It would be expected that the differences in the porcine tissue from which the lenticules are formed may be lesser than in human tissue across the general population as all lenticules were obtained from pigs of the same breed and therefore likely to be genetically more similar to one another (Zhang and Plastow, 2011), and in addition, due to farming procedures are likely to be of a similar age and reared in a similar environment prior to slaughter. However, some variation is likely and may be difficult to control. It is possible however, that the processing of the tissue results in an adequate increase in stiffness in all tissue, whether or not the response to the processes are equal, and a such all implants may be adequately effective for treating keratoconus. However, if the aim was to manipulate the stiffness of the cornea to achieve greater precision in terms of refractive changes in addition to preventing progression of keratoconus this may be a property that requires quantification, and interferometry may be a useful tool to enable this through mechanical pre-screening of the implants.

The findings of this pilot study are clear, however, due to the relatively small sample size, it would be helpful to conduct measurements on a larger number of samples to confirm these current findings. Furthermore, it would be useful to measure samples at different stages in the production process, for example; after removal from porcine cornea; post-decellularisation, post-compression and post-crosslinking to establish individually the effects of each of these processes on material stiffness and across individual corneas to ascertain where variability is likely to be introduced and whether greater repeatability in mechanical properties can be achieved. It would also be useful to use complimentary imaging techniques, such as two-photon microscopy (Steven et al., 2010) on the processed samples to quantify factors such as the degree of crosslinking to establish whether this correlates with the measured increase in stiffness. In addition to mechanical measures, to ensure suitability for implantation and long-term safety and effectiveness, it is important to characterise other properties of the lenticules, including, optical, thermal, and biological properties and to ascertain data on the stability of the properties and performance of the lenticules over their intended lifespan.

Ultimately, it is not only necessary to understand the biomechanics of the lenticule as a standalone material, but it is important to understand the effects that insertion of the implant has on the biomechanics, and subsequently refractive properties, of the cornea and how this changes over time. It is therefore important to investigate the biomechanics of the whole system at different stages of the recovery process and after long term implantation. This is something that is currently difficult to achieve due to a relative absence of measurement systems capable of carrying out a comprehensive analysis of corneal biomechanics. *in vivo*, as has been discussed extensively in the literature (Kling and Hafezi, 2017; Wilson and Marshall, 2020). Whilst DSPI can provide a useful evaluation of the mechanical properties of thin lenticules *ex vivo*, *in vivo* assessment is much more challenging and DSPI has several limitations, including high sensitivity to noise, poor tolerance for unwanted movements, poor signal to noise ratio in the absence of a corneal coating and the ability to obtain information only from the corneal surface. To understand the system as a whole, it is important to examine the 3-D stress and strain distribution through the thickness of the sample, this is especially important when considering the implantation of a lenticule as it is important to understand the biomechanics at the interface between the lenticule and neighbouring corneal tissue and how this changes over time. Several technologies capable of through thickness biomechanical assessment are currently under development for *in vivo* application, including optical coherence elastography (Kling et al., 2020), high frequency ultrasound (Pavlatos et al., 2018) and Brillouin spectroscopy (Shao et al., 2019) based-systems that may have useful application in this regard, however since all are scanning-based technologies comprehensive *in vivo* analysis of the type required here is challenging due to the requirement for long-scanning times to obtain adequate resolution data across the full cornea and the inherent issues this brings with regards to managing the effects of head and eye movement on data collection. A useful intermediate step may be to implant lenticules into corneas *ex vivo* and use DSPI (due to its advantages in obtaining high resolution, high sensitivity, full-surface deformation information in a single image) in combination with one of the aforementioned techniques capable of through-thickness assessment to understand the deformation patterns of the cornea to pulsatile pressure variations before and after insertion of the lenticule.

Through conducting these types of investigations it may be possible to gain an understanding of how to optimise both the mechanical properties of the lenticule and potentially the surrounding tissue through the use of adjunct and targeted therapies such as collagen crosslinking to increase quality of visual outcomes and long-term stability and biocompatibility.

Lenticule development is an on-going iterative process, therefore these experiments relate to Xenia^®^ implants at the time of experiments. Modifications are continuously being made to the processes used in-order to optimise their properties.

## Conclusion

The proprietary process used to produce Xenia^®^ corneal implants from porcine corneal tissue results in an average increase of Young’s modulus by 127% over unprocessed native porcine tissue when measured using DSPI under physiologically representative pressure fluctuations. There appears to be significant variability in the mechanical properties of both the control tissue and the processed lenticule, with variability across samples being significantly higher after processing. This requires further investigation to determine which part of the process results in this variation to allow more precise control.

Interferometry could potentially provide a means to quantify the mechanical properties of implants which could be useful in terms of developing and standardising their properties prior to clinical use and optimising the properties of lenticules to achieve the best long-term outcomes in terms of stability and bio-compatibility.

## Data Availability

The original contributions presented in the study are included in the article/Supplementary Material, further inquiries can be directed to the corresponding author.
