# Supplementary figures and images for: Biomechanical Evaluation of Decellularized and Crosslinked Corneal Implants Manufactured From Porcine Corneas as a Treatment Option for Advanced Keratoconus
Source: Front Bioeng Biotechnol. 2022 Apr 14;10:862969. doi: 10.3389/fbioe.2022.862969 (PMC9046912; doi:10.3389/fbioe.2022.862969)

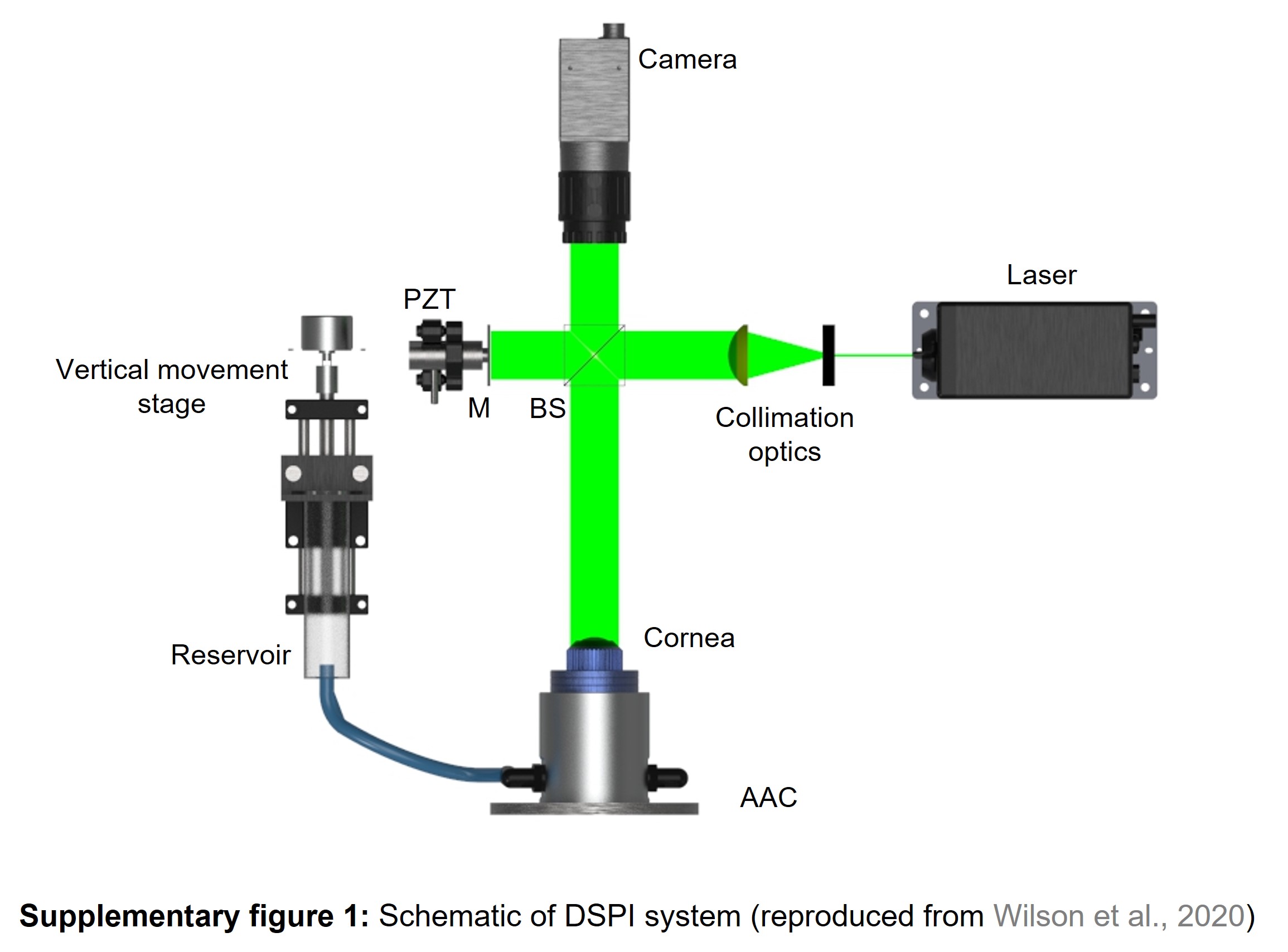

Supplement: Supplementary file 1 [file Image1.jpg]
